# Seabird species vary in behavioural response to drone census

**DOI:** 10.1038/s41598-017-18202-3

**Published:** 2017-12-20

**Authors:** Émile Brisson-Curadeau, David Bird, Chantelle Burke, David A. Fifield, Paul Pace, Richard B. Sherley, Kyle H. Elliott

**Affiliations:** 1 Department of Natural Resource Sciences, McGill University, Ste-Anne-de-Bellevue, Québec, Canada; 2 Psychology Department, Cognitive and Behavioral Ecology Program, Memorial University, St. John’s, NL Canada; 3 Wildlife Research Division, Environment Canada, 6 Bruce Street, Mount Pearl, NL Canada; 4 13 Erinlea Court, Ottawa, ON K2E 7C8 Canada; 5 Environment and Sustainability Institute, University of Exeter, Penryn Campus, Penryn, Cornwall, TR10 9FE UK

## Abstract

Unmanned aerial vehicles (UAVs) provide an opportunity to rapidly census wildlife in remote areas while removing some of the hazards. However, wildlife may respond negatively to the UAVs, thereby skewing counts. We surveyed four species of Arctic cliff-nesting seabirds (glaucous gull *Larus hyperboreus*, Iceland gull *Larus glaucoides*, common murre *Uria aalge* and thick-billed murre *Uria lomvia*) using a UAV and compared censusing techniques to ground photography. An average of 8.5% of murres flew off in response to the UAV, but >99% of those birds were non-breeders. We were unable to detect any impact of the UAV on breeding success of murres, except at a site where aerial predators were abundant and several birds lost their eggs to predators following UAV flights. Furthermore, we found little evidence for habituation by murres to the UAV. Most gulls flew off in response to the UAV, but returned to the nest within five minutes. Counts of gull nests and adults were similar between UAV and ground photography, however the UAV detected up to 52.4% more chicks because chicks were camouflaged and invisible to ground observers. UAVs provide a less hazardous and potentially more accurate method for surveying wildlife. We provide some simple recommendations for their use.

## Introduction

The number of organisms in a population has long been recognized as a key parameter in population ecology^1–3^. In most cases, it is not possible to completely census wild organisms and the estimated number of individuals is derived by correcting the sampled population to account for animals not sampled^4,5^. Furthermore, in remote areas, such as the Arctic or deep ocean, it is logistically difficult, expensive and hazardous to conduct wildlife surveys. In fact, boat and air accidents are the main cause of job-related mortalities for wildlife workers in the United States^6^. Unmanned aerial vehicles (UAVs), commonly referred to as drones, provide an opportunity to quickly sample more individuals in inaccessible environment, increasing the proportion of individuals observed and therefore the accuracy and precision of the population estimates, while improving security of biologists^7–11^.

While the potential benefits of UAVs are widely recognized^12–15^, the use of such vehicles also faces some potential drawbacks. First, early models required experienced operators, were prone to crashes and were often illegal even if widely used. The legality of unmanned aerial vehicle use has become clearer in many jurisdictions while technological advances have greatly simplified and improved operation^16^. Second, UAV observations may influence the behavior of wildlife and potentially bias the number of individuals counted^17,18^; the effect of UAVs on wildlife behavior has meant that wildlife agencies are reluctant to grant permits and that population estimates may be inaccurate. However, some wildlife show minimal reaction to unmanned aerial vehicles and/or habituate rapidly to their presence^19,20^, potentially allowing for accurate population estimates^21^. Given the growing use of UAVs by professional ecologists, amateur wildlife photographers and nature enthusiasts, guidelines for the ethical use of UAVs are increasingly needed^22^.

Colonial birds are one group of organisms where UAVs may be particularly suited for population surveys. Because many nest on flat ground or on cliffs, seabirds can be easily photographed and counted by a UAV. In contrast, the limited perspective of ground observers, usually standing in blinds, makes it difficult to fully census cliff-nesting species. Aerial surveys with human observers are another alternative, but can be dangerous and expensive, especially on remote islands where seabirds often occur. Recent work on terns (*Sterna* spp.) showed that unmanned aerial vehicles can provide less variable estimates of tern numbers^21^, and that terns quickly habituate to vehicles^23^. Similarly, another study on waterbirds showed little behavioral response to vehicles^22^.

To develop an effective method for counting cliff-nesting Arctic seabirds using unmanned aerial vehicles, we examined the effect of the vehicles on breeding seabirds and compared counts made from the vehicles with those made from observer-based photography. Environment and Climate Change Canada (ECCC) has a well-researched protocol for assessing changes in populations of *Larus* gulls and murres (*Uria* spp.)^24,25^. Those protocols have been used for over 40 years, and are based on counting standardized plots within colonies. However, because of the difficulty of accessing some locations, estimates for many colonies have not been completed since the 1970 s^25^. The ECCC protocol is based on correcting the number of birds present by accounting for the proportion of birds present likely to have laid at least one egg (breeding pairs). That correction factor is referred to as the k-ratio, and is ~0.7 for murres and ~1.0 for gulls^25^. Typically, the k-ratio varies with time of day because birds usually forage at the same time of day, and declines with date because young, non-breeding birds arrive later in the breeding season^25^. The k-ratio may also vary across the colony, as plots with many non-breeders (“loafing ledges”) have especially low k-ratios^25^. Continuing the earlier research on k-ratios developed from observer-based counts, we addressed a series of questions: How do seabirds respond to UAVs? Do they habituate, and how quickly? Does the approach direction or distance matter? Are UAV counts comparable to those derived from traditional census techniques and are they more or less variable?

## Methodology and Results

The study was conducted on thick-billed murres (*Uria lomvia*), a model-species for Arctic seabirds. In addition to the experiments on thick-billed murres, observations from three other surveys using UAVs as a secondary tool were added to this paper. These surveys were conducted on common murres (*Uria aalge*), on glaucous gulls (*Larus hyperboreus*), and on Iceland gulls (*Larus glaucoides*). As these differentiate from the main experiments on thick-billed murres for being opportunistic observations with low sample size, they should only be interpreted as additional considerations. To simplify the comprehension of these experiments, we separated the methodology and results for each species. All methods were approved through the Canadian Wildlife Service permit (permit number: NUN_SCI_16_03_Elliott) and the Wildlife Research permit emitted by the Department of Environment of the Government of Nunavut (permit number: 2016-036). All methods were performed in accordance with the Canadian Council of Animal Care norms and the Transport Canada legislations.

### Thick-billed murres

#### Methodology

Two arctic colonies were chosen for being the most accessible and studied colonies in Canada: Coats Island west murre colony (62°56′52.20″N, 82°01′03.70″W) and Digges Island colony (62°33′27.23″N, 77°43′18.20″W). We ran experiments and surveyed murres between 18–23 July 2016 at Coats Island and 28 July to 10 August 2016 at Digges Island, using a Phantom IV (DJI, Shenzhen, China), equipped with an in-built 12 M pixel camera (20 mm lens). At Coats, we estimated the effect of the vehicle on nest failure rate by monitoring six plots (each with 60 to 200 pairs of murres) daily for five days before and two days after one to five days of flights. Plot monitoring consisted of watching the murres from a blind and recording which individuals had an egg on each day^26^. Thus, daily nest failure rate could be calculated. As a polar bear (*Ursus maritimus*) eliminated all eggs on two plots prior to the first UAV flights, we eliminated those two plots from statistical analyses of reproductive success. Those two plots were kept in the analysis of murre’s response to UAV, as they represented a good opportunity to study plots with low k-ratio. All plots were less than 20 m from a blind.

**Figure 1:**
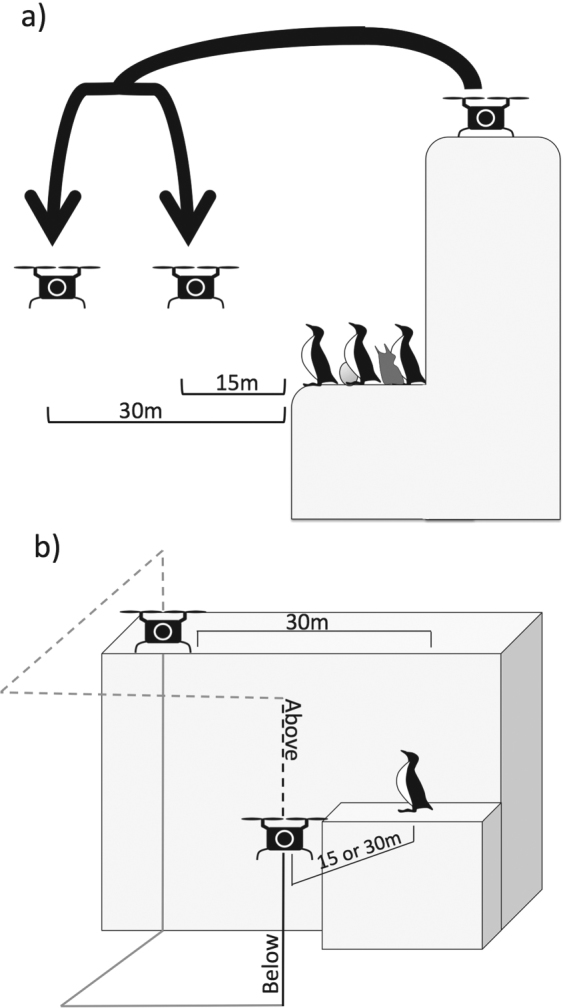
Representation of every approach of the plots with the UAV. (**a**) Take-off directly above the plot, with in-flight distance of 15 or 30 m to the plot. Birds with chicks or eggs are noted as breeders, while others are noted as non-breeders. (**b**) Take-off 30 m away from the plot. Emphasize of the two approaches possible: from above (dashed line) and from below (full line). The final darker lines for both methods represent when the birds are most likely to see the UAV. As with a), the UAV can stop at either 15 or 30 m to take the picture, and the ratio of non-breeder is also noted.

To examine the behavioral response of murres on these six plots to the UAV, we slowly flew the UAV toward the breeding plot until the horizontal distance from the plot was either 15 m or 30 m, let it hover long enough to take a picture, and then flew the UAV back (Fig. 1). The UAV started either on the edge of the cliff directly above the plot (~15–20 m above the plot) or 30 m further along the edge of the cliff. In the latter case, we also approached the plot either from above (flew the UAV up in the air, then brought it to 15 m or 30 m horizontal distance from the plot, then lower for the plot photo) or from below (lowered the UAV, brought it to 15 m or 30 m horizontal distance from the plot, then raised it for the plot photo). The sequence of flight patterns was randomly attributed. While the pilot was controlling the UAV, a second person recorded the number of birds flushing by videotaping (about 60 s) the plot from a blind and counting the number of birds leaving the colony using the recording. The ratio of birds flushing was then obtained by dividing with the total number of birds seen in the video. To examine habituation at small-scale, we repeated the previously described flights every five minutes for 4–6 flights on all six plots (and videotaped for one minute prior, during and one minute following each flight). We hypothesized that all flushed birds would come back to their nest within five minutes. On two plots, we repeated those same procedures for four and six consecutive days respectively, to examine habituation. Observers were invisible to birds at all times, either hidden behind the cliff face or in a blind. Assessment of the k-ratio prior to the flights were made by estimating the percentage of non-breeders in a plot. Birds without eggs or chicks were considered to be non-breeders.

We used the UAV to count 16 plots of ~30 to ~200 pairs. At Digges, we had the opportunity to repeat these flights four times per day (09:00, 12:30, 16:00, 20:00) to evaluate the repeatability over time. In addition to the counts using the UAV, birds were counted by taking a photograph (Panasonic fz-1000) and by sight, using the same plot-counting technique in use for Arctic monitoring over the past 40 years^27,28^. It took about two hours to count all plots once using both methods. This includes 15 minutes per plot for the UAV and 12 minutes for the ground method. It is important to note that the UAV method was not faster, due to the fact that we walked the UAV between each plot to standardize the method rather than scanning once all the plots with the UAV.

All statistical analyses were done in R 3.1.2 (R Core Team, 2014). The packages nlme (Pinheiro, Bates, DebRoy, Sarkar and R Core Team, 2016) and AICcmodavg (Mazerolle, 2016) were also used. Thirty linear models using 5 explanatory variables were created to analyze flushing intensity of thick-billed murres in response to a flying UAV. Flushing intensity was quantified as the proportion of murres flushing from a plot from the moment of takeoff to the moment of landing. To achieve normality and account for the bounded nature of proportion values, we logit-transformed proportion data prior to analyses. The explanatory variables were: the number of previous flights on the same day of the on-going observation (i.e. short-term habituation), take-off distance from the plot, in-flight distance from the plot, the average ratio of non-breeding birds in the plot (which are bound to react differently to approaching danger), and the angle of approach (either from above or from below). We also included interactions, based on the possible biological explanations. Models were ranked using the Akaike Information Criterion corrected for small sample size (AICc)^29^. Two variables were not included directly in the model, but rather in a separate analysis: 1- habituation over a long period (days) which was not included directly in the models, as high winds allowed us to only repeat the flights for three days or more at two plots 2- time of day, which could be consistently recorded at different time only in Digges Island, due to the conditions at Coats Island.

To examine the influence of UAVs on reproductive success, we used paired t-tests to compare site failure rates on days with UAV flights to days without UAV flights. The same test was also used to detect differences between ground counts and UAV counts relative to the time of the day. Prior to using parametric statistics, we tested for normality by inspecting the distribution of residuals from general linear models. We used an alpha level of 0.05. All averages are presented ± SD.

#### Results

**Figure 2:**
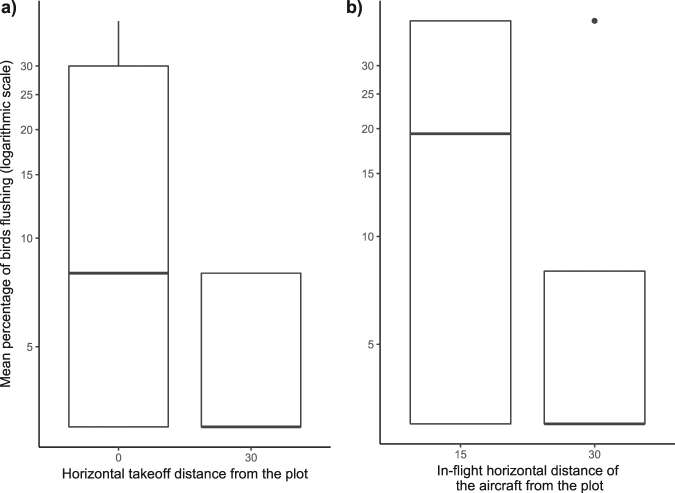
Boxplot of percentage of thick-billed murres flushing when (**a**) the UAV took off either 15–20 m directly above the plot at 0 m horizontal distance or 30 m away; (**b**) the UAV hovers at 15 m or 30 m from the plot surveyed. Y-axis was transformed with log10 to stretch the graph for visual purposes.

Take off distance, in-flight distance and the ratio of non-breeders on a plot were retained in the best linear model to explain the flushing behavior of murres. Angle of approach and all interaction terms were not retained in the best-fit models. As for habituation over a short period, it was present in the third ranked model, which had a small delta-AIC of 1.76 compared to the best-ranked model (Supplementary Table 2). However, its relative importance (RI) compared to the other three retained variables was low (0.20), suggesting this variable was not important for our model. The ratio of breeders was positively correlated with flushing (slope = 0.033 ± 0.005, RI = 1.0), while Take off distance (slope = −0.056 ± 0.008, RI = 1.0) and in-flight distance (slope = −0.026 ± 0.015, RI = 0.65) on the other hand, were negatively correlated with flushing (Fig. 2). On average, 8.5% of birds flushed. Most of those birds (>99%) that flushed did not have eggs and were thus considered non-breeders. Observations using the UAV thus had higher k-ratios. Five minutes after the series of flights, the majority of birds seemed to have returned to the plot, while >99% had returned after ten minutes. Flushing intensity stayed constant (SD = 0) within all plots monitored for several consecutive days and thus no habituation was detected. Nest failure rate averaged 0.11 ± 0.05% sites/d on days without vehicles compared with 0.09 ± 0.07% sites/d on days with UAV (t_3_ = 0.31; P = 0.78). We did not directly observe the loss of any egg within 1 hour following the vehicle flights.

**Figure 3:**
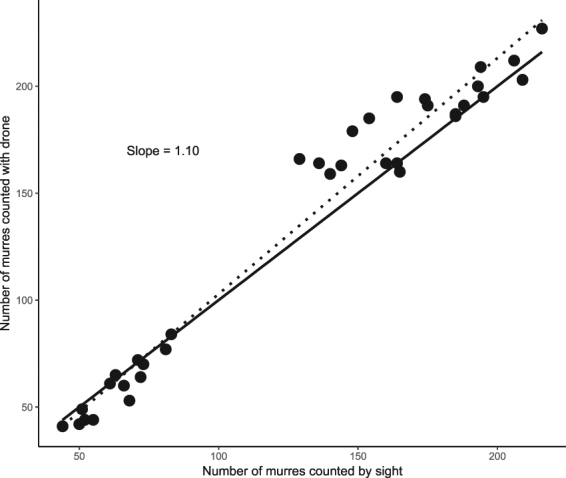
Comparing counts of thick-billed murres done by sight with counts done with the UAV. Dotted line represent the least squares regression while the filled line shows the line of 1:1 equality.

**Figure 4:**
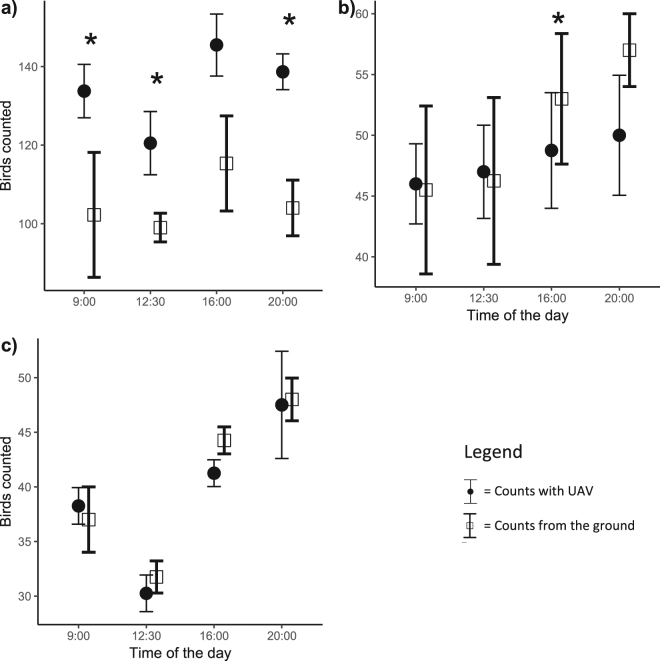
Thick-billed murres counted throughout the day from the ground and with the UAV, in (**a**) an example of very dense plot with few non-breeders (~0%), (**b**) an example of plot with low density, but with a considerable portion of non-breeders (~20%) (**c**) an example of plot with low density and few non-breeders (~0%). Stars indicate if a group is significantly different than its associated group for the same time of the day. Vertical bars represent 95% confidence intervals.

Counts were higher with the UAV than by sight, despite murres flushing from the plots when the UAV took off (t_69_ = 4.02; p < 0.01, see Fig. 3). The ratio of birds counted by UAV to those counted from the ground increased with density (t_38_ = 3.04, P < 0.01), and decreased with non-breeder density (t_69_ = −3.19, P < 0.01). In high-density plots (+100 birds), an aerial view with the UAV counted more birds, even if some murres flushed (Fig. 4a). In low density plots (−80 birds) with many non-breeders (+15%), counts were sometimes higher on the ground, especially at the end of the day, when proportionally more non-breeders were present at the colony. In these cases, few extra birds were counted with the better angle of the UAV and this could not account for the flushing birds (Fig. 4b). In low density plots with few non breeders (−10%), neither flushing nor aerial viewpoint were determinant factors, and counts on the ground (with camera or by sight) were similar to those using the UAV (Fig. 4c). Counts done on ground didn’t show more variability throughout the day, as the coefficient of variation (CV = 12.5%) was similar to the counts done by the UAV (CV = 11.6%).

### Glaucous gull

#### Methodology

Some scattered glaucous gull nests subsist within the thick-billed murre colony of Coats Island. Therefore, when flying the UAV for the thick-billed murre experiment, we also noted the behavior of glaucous gulls. Furthermore, as regular ground surveys of the glaucous gull population are conducted at Coats, we decided to use the drone to count all glaucous gull nests and chicks on 20 and 23 July 2016 for comparison with the ground surveys.

#### Results

We counted 16 nests and 20 chicks from the ground, compared with 16 nests (+0.0%) and 23 chicks (+15.0%) from the UAV. The adult glaucous gulls did not appear to respond to the UAV, whether it was during flights for the thick-billed murre experiment or for the glaucous gull survey. Thus, k-ratios were identical from site and UAV for gulls, but the number of chicks was underestimated by sight.

### Common murres

#### Methodology

Experiments on common murres were conducted at the sub-Arctic Gull Island, Newfoundland (47°09′36″N, 52°27′36″W). A Spyder X8 (SkyHero, Brussels, Belgium) was flown four times for reconnaissance between 19h30 and 20h30 on 18 June 2015 and 5 July 2016 at an altitude of 25 m above the ocean and a flight speed of 5 m/s, each separated by five minutes, at 80, 50, 30 and 25 m horizontal distance from the colony. We thus videotaped the murres to record their reaction to the UAV. On each flight, we flew past two subcolonies on the southeast side of the island, and estimated the number of birds flushing from the cliffs. In 2016, we established a study plot on June 29^th^ that included 40 breeding sites with incubating adults on the southwest side of the island. Over a four-hour observation period on June 30^th^, information relating to all observed flushing events were recorded including time of day, apparent cause of disturbance (gulls, observers, tour-boats, eagles), and total number of focal adults with eggs remaining on the study plot after each disturbance event.

#### Results

In 2015, 82% of murres remained after the first pass (80 m distant), followed by 81% (of the initial count) on the second pass (50 m), 83% on the third pass (25 m) and 78% on the fourth pass (20 m). We observed no birds with eggs leave the colony in response to the vehicle. In 2016, most birds frequently flushed when humans approached or made abrupt movements, even prior to UAV surveys. This year happened to be marked by the presence of four bald eagles (Haliaeetus leucocephalus) foraging near the colony. Therefore, many eggs were subsequently taken by herring (*L. argentatus*) and great black-backed (*L. maritimus*) gulls. Specifically, during baseline observations on 30 June, 12 large flushing events were observed over a 4-hour period (08:30–12:30), primarily attributed to gull disturbance (no eagles were seen). Disturbance involved widespread, synchronous flushing of non-breeding murres, but incubating murres did not abandon during any flushing event and no eggs were lost. During the single UAV flight, 93% of non-breeding birds within the established study plot flushed as the UAV approached the colony and 10% (4 of 40 active breeding sites) were lost. Adults were observed abandoning all four sites, resulting in egg displacement (n = 2) and subsequent egg predation (n = 2) by herring gulls. On 5 July 2016, the UAV was struck on the right front quarter with enough force to break the propellers by a single herring gull (*Larus argentatus*). This happened after passing the colony on the way back to land, about 75 m past the colony as it flew over gull nests with young chicks. The data telemetry logs indicate the UAV was working perfectly up to the point it was struck. The gull involved in the collision was seen flying away after impact, apparently unharmed. This incident ended the field experiment at Gull Island.

### Iceland gull

#### Methodology

One ground survey is organized annually at the Coats Island gull colony (62°48′13.33″N, 82°03′53.99″W) to count the Iceland gulls that nest in the area. We seized the opportunity to use the phantom IV to surveyed adults and chicks on 17 July 2016 for comparison. We flew the UAV three times for five to ten minutes at one subcolony and a single time at two other subcolonies.

#### Results

Across all subcolonies, we counted 92 birds, 57 nests and 21 chicks from the ground compared with 94 birds (+2.2%), 57 nests (+0.0%) and 32 chicks (+52.4%) with the UAV. The gulls immediately alarmed upon the arrival of the UAV 32 ± 5 m from the cliffs. However, all birds had returned to the cliffs within 3.3 ± 1.2 mins

## Discussion

Robots are increasingly being used in ecology^30–33^, and our study shows their promise for surveying wildlife. Using small, cheap (~$1600 US) rotary UAVs, we were able to accurately survey the number of breeding cliff-nesting birds at remote colonies, including one species of murre and two species of *Larus* gulls. Given that murres and gulls are well-known for their willingness to flush in response to human presence, we were surprised that breeding birds generally did not flush, and we could make accurate counts of the number of breeding birds on study plots. The only exception was in Newfoundland when the presence of eagles likely made the birds skittish, and a great number of breeding birds flushed in response to the UAV and lost their eggs. Thus, in contrast to an earlier study that found a similar response to UAVs among three species^22^, we found substantially different behaviors for the four species studied. While we show that UAVs can be used to accurately survey several species of birds, we also show that they can lead to substantial behavioral disruption and inaccurate counts when aerial predators are common. Vas *et al*.^22^ found that angles of attacks similar to those used by aerial predators caused greater flushing. We recommend pilot studies that examine behavioral responses prior to actual surveys, especially under conditions where the UAV may be perceived as an aerial predator.

Iceland gulls, which typically scold nest predators, were the most reactive at first to the UAV with most individuals flying off their nests. However, because all birds returned to their nest after about three minutes and counts with the UAV were accurate, we believe using UAVs is a reliable way to survey those cliff-nesting birds. Moreover, we counted more offspring with the UAV than from the ground, presumably because the offspring tended to hide behind boulders and were well camouflaged with the background. From the ground, it was often hard to view the nests except through a telescope from a great distance. The UAV could approach the nest within a few meters with little apparent response from the chick, and we could consequently search for the offspring with a high resolution image that could be tilted or moved in search of offspring. Apart from the incident with the herring gull in Newfoundland, no bird approached the UAV close enough to create high risk of collision, including glaucous gulls and peregrine falcons (*Falco peregrinus*) nesting among murres, even when it was 5–10 m from their nest. We believe a UAV approach is thus safe for both parties, as long as the UAV pilot has reasonable experience.

For non-scolding birds (murres), the flushing behavioral response was greater for non-breeders than for breeders. Breeding birds are tightly programmed to stay on their site for potential nest defense^34^, while non-breeders are not attached to a specific breeding site, and will easily fly away when disturbed. We were able to efficiently mitigate flushing for these birds by starting the UAV far from the plot, while in-flight distance reduced flushing by less than 4% as birds flushed primarily in response to the starting noise of the drone. Flying the UAV farther away from the plot to mitigate flushing also reduced image quality and increased time spent counting. Counting from a greater distance would require a better camera, with a larger lens and consequently a larger UAV. The larger UAV may cause more disturbance; McEvoy *et al*.^35^ noted that a larger UAV caused waterfowl to move rapidly away when approached closer than 40–60 m. We recommend that a distance of 20–25 m for surveying cliff-nesting birds with a small (2 kg) UAV. Future research could examine the trade-off between a larger UAV (larger, higher resolution camera), murre flushing and distance from the colony needed for sufficient resolution.

Angle of approach did not appear in the best-fit model explaining thick-billed murre flushing ratio. In contrast, Vas *et al*.^22^ found that vertical approaches caused substantially more flushing than oblique angle approaches for waterbirds in a flat wetland. For cliff-nesting birds, where a vertical angle of attack may be less associated with predation, angle of attack is apparently less important. *Larus* gulls habituated after ~3 min, so we recommend taking survey photographs after at least 3 min for those species to allow for accurate censuses of the number of adults, nests and offspring. In contrast, we found no evidence for habituation over short (min) or long (days) scales in murres, and so we do not advocate for multiple flights to reduce flushing. We also suggest that researcher take into account the presence of predators in the area before using UAVs, as bird response could be excessive in that case and a high level of nest desertion might be observed.

Mitigation of flushing by birds may however not always be ideal for surveys. Because population censuses are usually considered to be counts of breeding pairs^36,37^, the flushing of the 20% of non-breeders that occurred when we flew the UAV potentially reduces the coefficient of variation for the k-ratio, possibly leading to more accurate estimates. Similarly, counts of both temperate and tropical seabirds from UAVs averaged similar numbers to ground observers, but UAV counts were less variable due to reduced impact of geography and animal behavior on count numbers^21^. Much of the past efforts to provide accurate population indices for Arctic seabirds have focused on reducing variability in k-ratios so that counts can be easily converted into numbers of breeding pairs^38,39^. The proportion of non-breeders present is typically highest in the evening when pairs switch over their attendance duties, and when food availability is highest (late season in late-ice years, mid-season in early-ice years) and varies from year to year such that the proportion is high in years of high food availability^38,39^. By flushing many non-breeders, that source of variability is reduced by the UAV. The ability of the UAV to reveal otherwise hidden breeding birds from a ground perspective also adds accuracy to the counts.

Another advantage of the UAV is to quickly survey parts of the colony that are inaccessible. In the field, counts from the UAV for all three species could be completed by an observer stationed at one site in a few minutes, whereas ground counts typically took 1–2 hours for each species and required travelling considerable distances. Scaled up to an entire colony (~1 million birds), the time-saving could be dramatic. For instance, we photographed half of the Coats colony (~7500 breeding pairs) at close range in about 4 h (EBC, unpubl. data). This operation can take days in inclement weather if the ice isn’t secure for ground photos from the sea^40^. A UAV survey would then not only take less time, but may also be more accurate and less expensive. Moreover, of the 20 days we attempted to make censuses in 2016, we were only unable to fly on two days, although on several other days we could only survey sheltered areas of the colony. In Newfoundland, we were able to fly only a few hours out of the 10 days we had planned for surveys, mainly because of strong winds. Such issues could be overcome by using a fixed wing UAV that could fly in a larger range of wind conditions.

While we have focused on whether or not accurate surveys are possible, additional ethical concerns must be addressed for amateur photographers or enthusiasts. Repeated visits by humans are known to cause stress to wild animals, even if no obvious behavioral signs are observed. For example, bears showed strong physiological responses to UAVs even when there were seldom behavioral changes^18^. Additional research on heart rate or other components of stress beyond flushing in birds could provide additional information to guide ethical guidelines for the use of UAVs.

We provide the following recommendations for the use of UAVs to survey cliff-nesting seabirds. Those recommendations are likely a strong starting point for surveying other wildlife:

1. Given the species-specific responses, baseline tests are essential to determine whether wildlife disperse in response to UAVs, especially where avian predators are common.
2. For gulls, a period of 5 min for habituation to small rotary UAVs is essential prior to counts.
3. For murres, small rotary UAVs should be flown at a distance of at least 20 m and with a take-off outside of the hearing range of the colony. The UAVs are nonetheless likely to cause flushing in plots with high levels of non-breeders.

These recommendations apply mostly to small UAVs (<2 kg), similar to those used in the studies. We recommend that further research be conducted in the subject. More precisely, we suggest that future research focus on what exactly in the rotary-winged UAV (sound of wings, shape, etc.) causes bird to flush. We also recommend that further research compare the rotary-winged UAV with the fixed-wing UAV, as the latter might serve as an alternative in stronger winds.

In conclusion, our study adds to the recent but growing body of literature that measure the reaction of wildlife to UAVs, and illustrate that, if used appropriately, UAVs can accurately survey wildlife without excessive disturbance^16,22,34,41^. Our results on cliff-nesting birds could be extrapolated outside the Arctic ecosystem. In difficult-to-access habitats, such as marshes, UAVs could be a better option for surveying populations than traditional methods. Although the camera resolution for small drones is only capable of detecting large birds and mammals from a distance, the rapid improvements in UAV technology promise to open new avenues for accurately surveying wildlife in remote habitats. Given the variation we observed among species, we recommend that preliminary work examining the level of disturbance to wildlife is undertaken before comprehensive surveys are launched, especially for species that experience substantial predation by other birds.

## Electronic supplementary material

Supplementary Material
