# Supplementary material for: Seabird species vary in behavioural response to drone census
Source: Sci Rep. 2017 Dec 20;7:17884. doi: 10.1038/s41598-017-18202-3 (PMC5738335; doi:10.1038/s41598-017-18202-3)
Supplement: Supplementary file 1 — Supplementary Material [file 41598_2017_18202_MOESM1_ESM.pdf]

Seabirds species vary in behavioural response to drone census

Émile Brisson-Curadeau* [1,2], David Bird[1], Chantelle Burke[3], David A Fifield[4], Paul Pace[5], Richard B Sherley[6] and Kyle H Elliott[1]

[2]Corresponding author. Email: emile.brissoncuradeau@mcgill.ca

Phone: 1-514-553-6910

[1]Department of Natural Resource Sciences, McGill University, Ste-Anne-de-Bellevue, Québec, Canada

[3]Cognitive and Behavioral Ecology Program, Psychology Department, Memorial University, St. John's, NL, Canada

[4]Wildlife Research Division, Environment Canada, 6 Bruce Street, Mount Pearl NL, Canada

[5]13 Erinlea Court, Ottawa, ON K2E 7C8, Canada

[6]Environment and Sustainability Institute, University of Exeter, Penryn Campus, Penryn, Cornwall, TR10 9FE, UK

Supplementary Information

Table S1: Structure of 30 linear models explaining the ratio of thick-billed murres flushing from a plot in response to the UAV. The 6 explanatory variables are: 1) habituation over a short period of time, as the number of previous flights on the same day of the on-going observation 2) take-off distance from the plot in meters, 3) in-flight distance from the plot in meters, 4) the ratio of non-breeding birds in the plot (continuous), 5) the angle of approach (from above or from below). 6) Interaction from effects 1 and 3, 7) Interaction from effects 3 and 4. Models were built based on the relevant biological explanations.

	**(1) Habituation over a short period**	**(2) Take-off distance from the plot**	**(3) In-flight distance from the plot**	**(4) Ratio of non-breeding birds in the plot**	**(5) The angle of approach**	**(6) Interaction: Effects (1) and (3)**	**(7) Interaction: Effects (4) and (3)**
**m1**		x					
**m2**		x	x				
**m3**		x	x	x			
**m4**			x				
**m5**			x	x			
**m6**				x			
**m7**							
**m8**	x	x	x	x			
**m9**		x		x			
**m10**	x	x		x			
**m11**		x			x		
**m12**		x	x		x		
**m13**		x	x	x	x		

**m14**			x		x		
**m15**			x	x	x		
**m16**				x	x		
**m17**					x		
**m18**	x	x	x	x	x		
**m19**		x		x	x		
**m20**	x	x		x	x		
**m21**	x	x	x	x	x		**x**
**m22**			x	x	x		**x**
**m23**		x	x	x	x		**x**
**m24**	x	x	x	x			**x**
**m25**			x	x			**x**
**m26**		x	x	x			**x**
**m27**	x	x	x	x		**x**	
**m28**	x	x	x	x	x	**x**	
**m29**	**x**	**x**	**x**	**x**		**x**	**x**
**m30**	**x**	**x**	**x**	**x**	**x**	**x**	**x**

Table S2: AICc table for results of all models explaining the ratio of thick-billed murres flushing from a plot in response to the UAV

	K	AICc	ΔAICc	AICc Wt	Cumulative	Log
m3	6	169.66	0.00	0.25	0.25	-78.12
m9	5	170.22	0.57	0.19	0.44	-79.61
m8	7	171.41	1.76	0.10	0.54	-77.74
m13	7	171.73	2.07	0.09	0.63	-77.90
m26	7	172.15	2.49	0.07	0.70	-78.11
m19	6	172.21	2.55	0.07	0.77	-79.39
m10	6	172.55	2.89	0.06	0.83	-79.56
m27	8	173.57	3.91	0.04	0.86	-77.52
m18	8	173.70	4.05	0.03	0.89	-77.59
m24	8	173.99	4.33	0.03	0.92	-77.73
m23	8	174.33	4.67	0.02	0.95	-77.90
m20	7	174.66	5.01	0.02	0.97	-79.37
m28	9	175.89	6.23	0.01	0.98	-77.34
m29	9	176.26	6.60	0.01	0.99	-77.52
m21	9	176.39	6.73	0.01	0.99	-77.59
m30	10	178.66	9.01	0.00	1.00	-77.33
m2	5	180.13	10.47	0.00	1.00	-84.56
m12	6	181.71	12.06	0.00	1.00	-84.15
m1	4	182.32	12.67	0.00	1.00	-86.83
m11	5	183.44	13.79	0.00	1.00	-86.22
m16	5	189.93	20.27	0.00	1.00	-89.47
m15	6	191.67	22.01	0.00	1.00	-89.12
m22	7	194.16	24.51	0.00	1.00	-89.12
m17	4	197.29	27.63	0.00	1.00	-94.32
m14	5	198.94	29.28	0.00	1.00	-93.97
m6	4	204.19	34.53	0.00	1.00	-97.77
m5	5	205.92	36.26	0.00	1.00	-97.46
m25	6	206.83	37.17	0.00	1.00	-96.70
m7	3	213.73	44.07	0.00	1.00	-103.67
m4	4	215.74	46.08	0.00	1.00	-103.54
